# The prevalence of psychosis in epilepsy; a systematic review and meta-analysis

**DOI:** 10.1186/1471-244X-14-75

**Published:** 2014-03-13

**Authors:** Maurice J Clancy, Mary C Clarke, Dearbhla J Connor, Mary Cannon, David R Cotter

**Affiliations:** 1 Department of Psychiatry, Royal College of Surgeons in Ireland, Education and Research Centre, Beaumont Hospital, Dublin 9, Ireland; 2 Department of Psychiatry, Beaumont Hospital, Dublin 9, Ireland; 3 Department of Psychology, Division of Population Health Sciences, Royal College of Surgeons in Ireland, Beaux Lane House, Dublin 2, Ireland

**Keywords:** Psychosis, Epilepsy, Systematic review

## Abstract

**Background:**

Epilepsy has long been considered to be a risk factor for psychosis. However there is a lack of consistency in findings across studies on the effect size of this risk which reflects methodological differences in studies and changing diagnostic classifications within neurology and psychiatry. The aim of this study was to assess the prevalence of psychosis in epilepsy and to estimate the risk of psychosis among individuals with epilepsy compared with controls.

**Methods:**

A systematic review and meta-analysis was conducted of all published literature pertaining to prevalence rates of psychosis in epilepsy using electronic databases PUBMED, OVIDMEDLINE, PsychINFO and Embase from their inception until September 2010 with the following search terms: prevalence, incidence, rate, rates, psychosis, schizophrenia, schizophreniform illness, epilepsy, seizures, temporal lobe epilepsy.

**Results:**

The literature search and search of reference lists yielded 215 papers. Of these, 58 (27%) had data relevant to the review and 157 were excluded following a more detailed assessment. 10% of the included studies were population based studies. The pooled odds ratio for risk of psychosis among people with epilepsy compared with controls was 7.8. The pooled estimate of prevalence of psychosis in epilepsy was found to be 5.6% (95% CI: 4.8-6.4). There was a high level of heterogeneity. The prevalence of psychosis in temporal lobe epilepsy was 7% (95% CI: 4.9-9.1). The prevalence of interictal psychosis in epilepsy was 5.2% (95% CI: 3.3-7.2). The prevalence of postictal psychosis in epilepsy was 2% (95% CI: 1.2-2.8).

**Conclusions:**

Our systematic review found that up to 6% of individuals with epilepsy have a co-morbid psychotic illness and that patients have an almost eight fold increased risk of psychosis. The prevalence rate of psychosis is higher in temporal lobe epilepsy (7%). We suggest that further investigation of this association could give clues to the aetiology of psychosis.

## Background

The nature of the relationship between psychosis and epilepsy has been of great interest to psychiatrists for over a century [1-12]. In one of the most epidemiologically complete studies involving direct patient interviews of all individuals with epilepsy in Iceland, Gudmundson (1966) reported a rate of psychosis of 7.2% [13]. In keeping with this the majority of studies [3,4,14,15] have found a higher prevalence of psychosis in patients with epilepsy compared with the general population, but this finding is not consistent and reported rates varying from 0.48% [16] to 35.7% [17]. Methodological differences such as changing diagnostic classifications, clinical heterogeneity, different ascertainment methods, and lack of power likely account for much of the inconsistency. Previous qualitative reviews of this topic these reported an overall rate of psychosis in epilepsy of 7% and 7.6% respectively [18,19]. More recently Gaitatziz et al. looking at psychiatric morbidity overall in epilepsy more recently, estimated that the prevalence of psychosis in population based studies at between 2-7% and estimated a prevalence of psychosis at 10-19% in patients with TLE or refractory epilepsy [12].

Improving our understanding of the basis to the relationship between psychosis and epilepsy is important as it may provide clues to the pathophysiology of psychosis generally [4,20,21]. In the current investigation we have undertaken the first systematic review of the prevalence of psychosis in epilepsy. Our main aim was to estimate the overall pooled prevalence of psychosis among patients with epilepsy. Secondary aims were 1) to examine the prevalence of psychosis associated specifically with temporal lobe epilepsy, 2) to estimate the prevalence of postictal and interictal psychosis, 3) to estimate prevalence of psychosis in specific subgroups of patients with intellectual disability, children and adolescents, and individuals with genetic vulnerability to psychosis and 4) to carry out separate analysis for studies with control groups to allow estimation of risk for psychosis among patients with epilepsy in terms of pooled odds ratios.

## Methods

### Study selection

We conducted a systematic review of all published literature in English on the prevalence of psychosis in patients with epilepsy. We were guided by PRISMA criteria[22]. A search was undertaken of the electronic databases PUBMED, OVID MEDLINE and EMBASE from their inception to September 2010 with the following terms: prevalence, incidence, rate, rates, psychosis, schizophrenia, schizophreniform illness, epilepsy, seizures and temporal lobe epilepsy. In addition to this search procedure, we used the reference lists of the identified publications to find further relevant articles.

#### Inclusion criteria

Papers were included if they gave prevalence rates of psychosis, schizophrenia, interictal psychosis (see definition below) or postictal psychosis (see definition below) in persons with epilepsy. We included studies involving adults, children and patients with learning disability.

The definition of interictal psychosis (also known as the schizophrenia-like psychosis in epilepsy) is a psychotic disorder that would fulfill diagnostic criteria for schizophrenia if epilepsy was not present. The interictal psychotic disorders are of reasonably long duration and are not related to the occurrence of seizures [4,23].

The definition of postictal psychosis is a psychosis that manifests itself immediately upon a seizure or emerges with one week of return of apparently normal mental function .The psychosis has to last a minimum length of 24 hours and a maximum length of 3 months [23].

#### Exclusion criteria

Papers excluded were those that (a) did not have the full article published in English, (b) did not report prevalence rates or data from which rates could be calculated (c) involved case reports, letters, short reports, reviews or book chapters.

#### Data extraction

Following initial searches, all titles of papers and abstracts were examined and assessed for relevance and appropriateness of the main question under review. Full texts of potentially relevant papers were obtained. Authors of papers were contacted where necessary. The methodological quality of studies was assessed.

For the purpose of the meta-analysis, we extracted the following domains or variables from the articles that were finally included:

1) prevalence rates for psychosis including interictal and postictal psychosis and schizophrenia. (where specified)

2) date of publication, country (developing or developed world), year of publication, proportion of children or individuals with learning disability within epilepsy sample.

3) sample size, gender composition, whether the study was carried out in a hospital or community setting, study design (cross-sectional or cohort),

4) diagnosis of epilepsy and whether International League Against Epilepsy (ILAE) classification was used

5) diagnostic tools used for psychosis and method of obtaining diagnosis- (unstructured interview, case note review, structured interview).

### Data analysis

Pooled estimates of the prevalence of psychosis in epilepsy patients were calculated using random-effects meta-analysis. This allows a more robust and true estimate of effect size and one that is weighted by the sample size of individual studies. A random effects model weights studies more equally and is considered more appropriate for meta-analyses with substantial heterogeneity [24,25] and this meta-analytic approach has been used with data containing similar levels to those seen here [25]. The between-study variance or heterogeneity in estimates was modelled using Cochran Q and the I^2^ statistic. The Q statistic is reported with χ^2^ and p-values and the I^2^ statistic is reported as a percentage with increasing values indicating greater heterogeneity between estimates of individual studies (I^2^ <25% indicates low heterogeneity; 30-70% = moderate heterogeneity and >75% indicates high heterogeneity [25]. Meta-regression was used to estimate the extent to which measured covariates (study design, study setting, assessment instrument used, types of epilepsy, year of study, family history of psychiatric illness, International League Against Epilepsy (ILAE) classification used, exclusion criteria applied, country in which study took place) could explain the observed heterogeneity in prevalence estimates across studies. The regression coefficients (β) reported indicate the average difference in prevalence proportion for one category compared to the other (eg assessment by clinical interview versus case notes). Effects of individual covariates were examined first in univariate models and then in a multivariate model constructed in a step-wise fashion. All analyses were carried out using STATA statistical software package, version 11.0.

## Results

### Literature search

Our preliminary search identified a total of 431 papers on EMBASE, 773 papers on PUBMED and 999 papers on MEDLINE via OVID. There was a substantial degree of overlap between the 3 databases.

**Figure 1:**
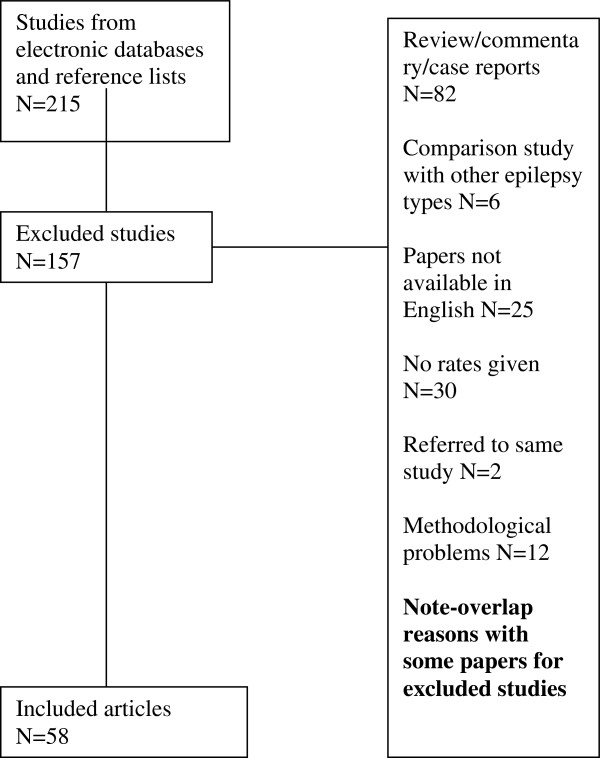
PRISMA (Preferred Reporting Items for Systematic Reviews and Meta Analyses) flow diagram.

After these titles were screened, 215 papers were examined in detail. After applying inclusion and exclusion criteria, 58 papers (27%) were deemed to have data relevant to the systematic review and meta-analysis. See Figure 1.

### Included studies and study design

Thirteen of the studies were from the UK, 11 from the US, 11 from Japan, 5 from Brazil, 2 from Denmark, 2 from the Netherlands, 2 from Canada, 2 from India,2 from Iceland, 2 from Australia and 6 from other countries.

Ten papers specified the gender of participants in the study. The number of patients with psychosis in the studies varied from 1 [16,26-28] to 795 [29].

The number of participants in studies varied from 50 [30] to 34,494 [29]. With regard to the prevalence rates, forty-eight studies were cross sectional studies, 9 were cohort and 1 was case control. The time periods over which the prevalence rates were calculated were as follows: 27 studies were for an unknown time period, 7 studies were lifetime prevalence studies, 21 studies were for more than 1 year and 3 studies were less than 1 year. Only 6 of the studies had control groups [31-36].

Seven studies were in a community setting, 3 were in a mixed hospital/community setting, 1 was unknown, 3 were from population databases and the remaining 44 were from hospital settings.

In 39 of the studies, patients were interviewed, in 19 studies the patients were not interviewed or it was not stated in the study.

The prevalence rates of psychosis varied from 0.02% [37] to 27% [17]. Forty of the studies used ICD criteria or DSM criteria for diagnosis. Of these studies, 17 used DSM IV criteria, 5 used DSM IIIR criteria, 1 used DSM III criteria, 1 used DSM II criteria, 6 used ICD 10 criteria, 4 used ICD 9 criteria and 1 used ICD 8 criteria. 5 studies used a mixture of these criteria.

Seven studies used a Structured Clinical Interview for DSM IV on patients in their studies [26,31,38-42]. Nine studies were conducted before 1990.

Seventeen of the 58 studies gave the prevalence of schizophrenia. Ten studies had prevalence rates for interictal psychosis. Thirteen studies had prevalence rates for postictal psychosis. Three studies used a sample group of patients with children/adolescents only [14,27,43]. Four studies studied patients with Learning Disability only [32,44-46]. Seventeen studies included patients with TLE only. Seven of the studies mentioned a family psychiatric history, only 3 mentioned a family history of psychosis. 17 studies had TLE patients only, in 26 studies, the authors did not differentiate whether patients had TLE or not and in 15 studies, there was a mixture of patients with TLE and other forms of epilepsy but unfortunately it was not possible to extract the non TLE or TLE sample from each other. Therefore for the TLE prevalence sample, we only used studies where all the patients had TLE. We did not contact authors personally to access data as this was not possible in many of the studies as the contact details were not current and 22 of the studies were conducted before 2000.

### Prevalence of psychosis in epilepsy

**Figure 2:**
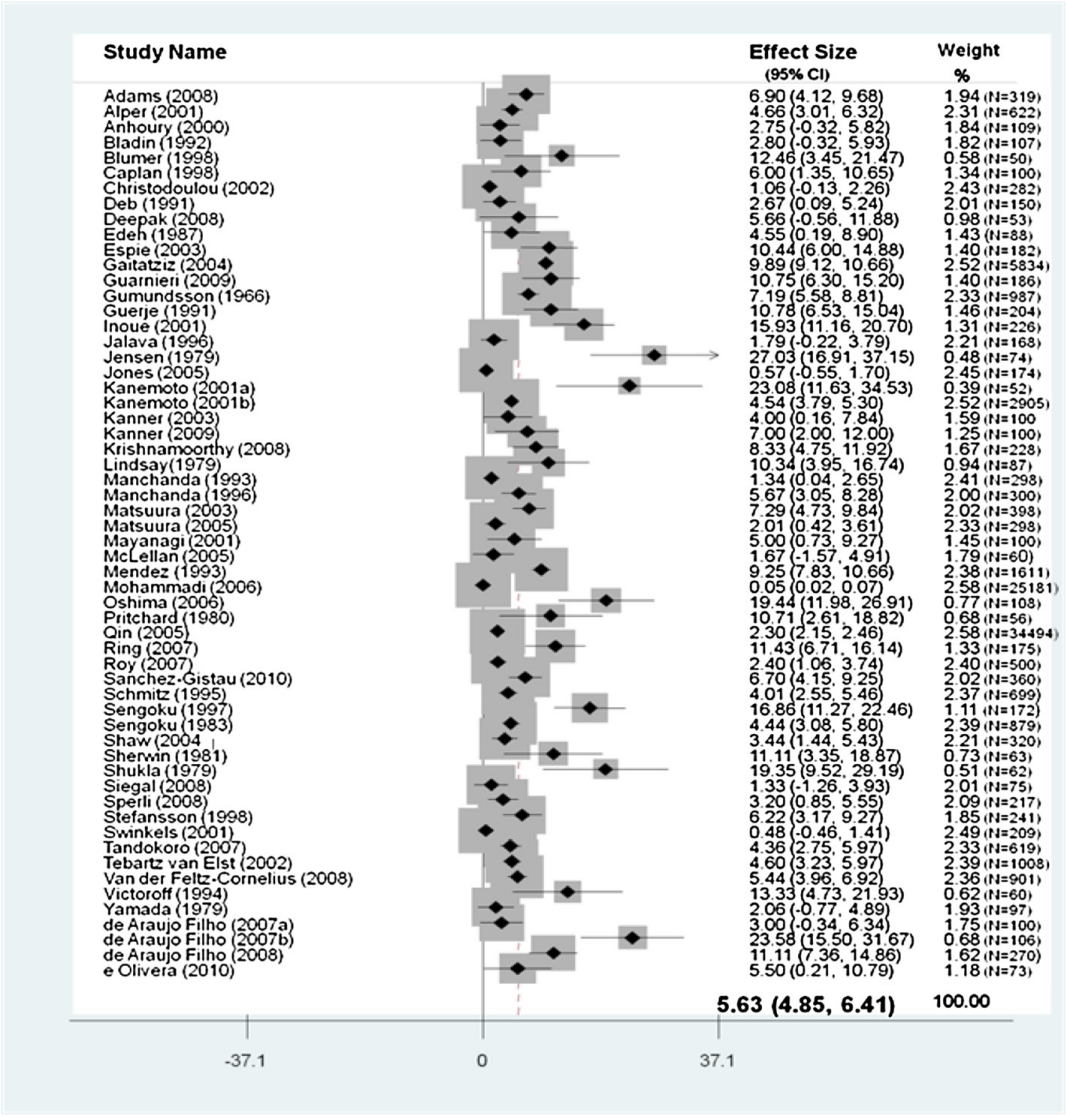
Pooled prevalence of psychosis in individuals with epilepsy.

Overall, the pooled prevalence rate for psychosis in epilepsy patients was 5.6% (95% CI =4.8-6.4). (see Figure 2). The pooled prevalence rate when postictal psychosis was excluded was 5.4% (95% CI = 4.5-6.2).

**Figure 3:**
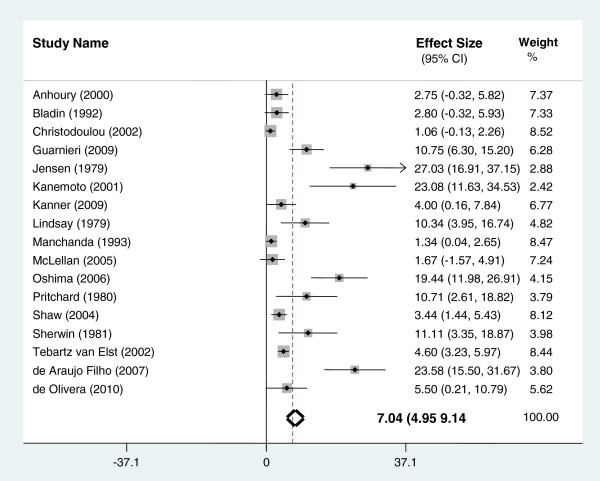
Prevalence of psychosis in temporal lobe epilepsy.

The pooled prevalence rate for psychosis in patients with temporal lobe epilepsy was 7.0% (CI 4.9-9.1). (See Figure 3). The pooled prevalence rate of interictal psychosis was 5.2% (95% CI 3.2-7.2) and the pooled prevalence rate for post ictal psychosis was 2.0% (95% CI 1.2-2.8).

Four papers studied an intellectual disability sample only [32,44-46]. The prevalence of psychosis in patients with epilepsy and comorbid learning disability was 7.4% (95% CI 2.6-12.2).

Three studies used child and adolescent subjects only [14,27,43]. The prevalence of psychosis in children and adolescents with epilepsy was 5.4%, (95% CI 0.6-10.2).

Three papers mentioned a family history of psychosis. The prevalence of psychosis in patients with epilepsy where a family history of psychosis was present was 5.4% (95% CI = 1-9.8).

**Figure 4:**
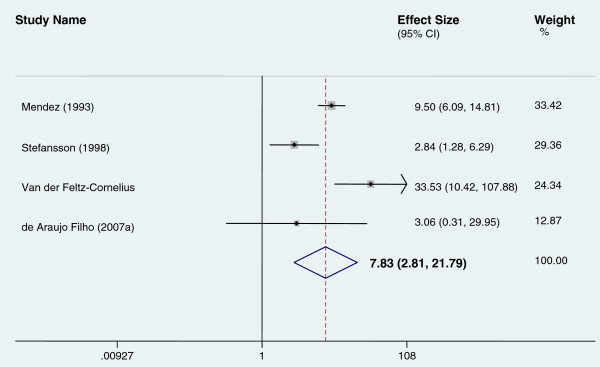
Pooled odds ratio for studies with controls.

Six of the studies had control groups. However, only 4 studies [31,34-36] were used for analysis because two of them [32,33] had no psychosis outcome in the control group so an odds ratio could not be calculated. The pooled odds ratio for risk of psychosis among people with epilepsy was 7.83. (See Figure 4).

### Heterogeneity analyses

There was substantial heterogeneity between the prevalence estimates of psychosis in epilepsy patients across individual studies (I^2^ > 70%). We examined the effect of nine factors on the prevalence of psychotic symptoms in epilepsy patients found across studies in a meta-regression analysis: (1) study design (2) study setting (3) instrument used to assess psychotic symptoms (4) types of epilepsy (5) year of study (6) family history of psychosis noted (7) ILAE classification used (8) exclusion criteria applied (9) country in which the study took place.

### Meta-regression analyses

None of the following factors significantly explained any of the variance in the estimates on an individual basis. (1) Study Design: (β = -1.1,SE(β = 1.9, p =0.58). (2) Study setting: (whether recruiting patients from the community or from hospitals or from tertiary centres) = (β -0.11, SE = (β) = 0.9, p =0.90). (3) Instrument used to assess psychosis: (employing a clinical interview or using case notes) (β = 0.31, SE(β) = 1.7, p =0.85). 4) Types of epilepsy reported: (all types of epilepsy patients or temporal lobe epilepsy patients only or other types of epilepsy) = (β = 0.74, SE(β) = 0.8, p =0.39). 5) Year of study: (whether studies carried out before 1990 or after 1990) (β = -2.8, SE (β) = 2.1, p =0.17). 6) Family history of psychosis: family of history of psychosis assessed or not assessed = (β = 3.7, SE(β) = 2.1, p =0.09).

7) ILAE classification used: studies reporting use of ILAE classification or not reporting this classification = (β = 0.3 , SE (β) = 1.7, p =0.85). 8) Exclusion criteria applied: studies reporting the use of exclusion criteria or those not reporting exclusion criteria (β = 1.1, SE(β) = 1.4, p =0.45). 9) Country in which study took place: studies from countries classified as ‘developed’ or those classified as ‘not developed’ (β = 2.0, SE(β) = 1.9, p =0.31).

A multivariate model constructed in step-wise fashion showed that the specific combination of 5 factors explained the largest amount of heterogeneity in the prevalence estimates: Study setting; Assessment instrument used; Year of study, Family history of psychosis; Type of epilepsy. These 5 variables together explained 10% of the variance in estimates of the prevalence of psychotic symptoms in epilepsy patients across studies.

## Discussion

This is the first systematic review and meta-analysis to examine the prevalence rate of psychosis in epilepsy. Our finding of 5.6% pooled prevalence of psychosis in epilepsy is lower than estimates of approximately 7% estimates from previous narrative reviews, [18,19] although our finding of 7% for the rate of psychosis in temporal lobe epilepsy is similar. Based on results from population based studies with control samples, [31,34-36] we found that the rate of psychosis among people with epilepsy is 7.8 times higher than in people without epilepsy. However there were very few studies fulfilling these criteria. Our findings are consistent with Gudmundsson who found that 7.2% of subjects with epilepsy were currently or had at some time suffered from psychotic illness [13].

We found a low prevalence of postictal psychosis in epilepsy-2%. This has traditionally been described as the most common form of psychosis in patients with epilepsy [23]. Prevalence rates of around 6% from two telemetry series have been reported in the past [39,47]. The relatively low postictal rate found in this study may be explained by the transitory nature of PIP, it’s acute and short presentation and that it is often managed in non specialised departments and may go undiagnosed. It may be mistaken for acute confusional states rather than postictal psychosis. However, postictal psychosis may be over or under represented depending on the availability of psychiatric assessment, the frequency of review and potentially the knowledge base of the treating neurologist and neuropsychiatry teams. The rate of interictal psychosis at 5.2% was over two and a half times the rate of postictal psychosis, but similar to the rate for overall psychosis in epilepsy.

TLE is the most common of the anatomically defined syndromes accounting for around 60% of all patients with localisation related epilepsies [23]. It is the most common type of epilepsy in adults who experience seizures poorly controlled by anticonvulsant medication. Our results found a slighter higher rate of psychosis in TLE as compared to all epilepsies (7% v 5.6%). There has been some debate in the past whether there is a higher rate of schizophrenia-like-psychosis in patients with TLE compared to generalized epilepsies. In the original study by Slater et al., the high rates of psychosis may have been in part due to somewhat imprecise clinical diagnostic data as the terms schizophrenia and psychosis were relatively loosely defined by today’s standards [4]. There is also the possibility of an ascertainment bias as the study drew its subjects from tertiary centres in two major London hospitals. More recently, Stevens argued that the proportion of TLE in epilepsy-psychosis patients is similar to the proportion rate of generalized epilepsy patients with epilepsy psychosis which is estimated to be about 60% [48-50]. Furthermore, in the large epidemiological study by Qin et al., patients with localization related epilepsy were only slightly over-represented among those who were psychotic and this difference fell short of statistical significance [29]. Several studies have failed to confirm the commonly held view that there is a specific association between temporal lobe epilepsy and psychopathology which is in contrast to commonly accepted clinical practice [51-53].

### Methodological issues

Most of the studies included in this review were cross sectional in design (77.6%) and based on samples from tertiary referral centres (71%) and these factors may limit the interpretation of the results with respect to the general population. Only 10% of the studies were population-based. Prevalence measures derived from such unrepresentative samples may therefore overestimate psychiatric morbidity among epilepsy patients [54].

We were unable to draw conclusions about the influence of a genetic vulnerability to psychosis from this study as data was too limited. Family history of psychosis was only mentioned in 3 studies [29,34,39]. Further family studies are needed to elucidate this association. Heterogeneity may also play a role.

It was not possible to identify a group of patients which did not have temporal lobe epilepsy to compare with the TLE group as some studies did not state whether the patients had TLE or not and also some studies has TLE and non TLE patients mixed together in their study samples, thus it was impossible to differentiate these 2 disparate groups out.

### Temporality of the association between epilepsy and psychosis

The question of whether epilepsy is a risk factor for psychosis and/or whether psychosis is a risk factor for epilepsy has repeatedly arisen in the literature, however, very few studies had the data necessary to adequately provide answers [55]. We have not addressed the temporality of the association in this study because the majority of studies included do not have temporal information. However, Clarke et al. have recently found in a population based family study that patients with epilepsy have a 5.5 fold increase in the risk of having a broadly defined psychotic disorder and an 8.5 fold increase in the risk of having schizophrenia [56]. Individuals with a parental history of epilepsy had a 2 fold increase in the risk of developing psychosis, compared to individuals without a parental history of epilepsy. Individuals with a parental history of psychosis had reciprocally a 2.7 fold increase in the risk of having a diagnosis of generalised epilepsy compared to individuals without a parental history of psychosis. Post hoc analyses showed that these analyses were not driven by the comorbidity of epilepsy and psychosis in the parents.

### Classification of psychosis

A consensus on the classification of psychotic symptoms associated with epilepsy is lacking [57]. Neither ICD 10 nor DSM V classify seizure-related psychosis separately. It could be debated whether postictal psychosis for example should be classified as a brief psychotic disorder/psychotic disorder due to a general medical condition or a psychotic disorder not otherwise specified in DSM. Furthermore, the psychopathology of patients with epilepsy can be atypical and does not readily conform to these diagnostic manuals [58]. A dedicated sub-commission of the International League Against Epilepsy commission on neuropsychiatric aspects has developed a proposal on the classification of neuropsychiatric disorders in epilepsy [59]. Recent genetic studies reinforce the view that more attention should be given to the relationship between the functional psychoses and neurodevelopmental disorders such as autism [60].

### Possible mechanisms

It has been suggested that the neurotoxic effect of epilepsy explains the association between epilepsy and psychosis [55]. Various mechanisms through which this effect might be brought about have been proposed. These include firstly, a ‘kindling’ process whereby acute seizure discharges may cause changes in brain function perhaps through receptor-based changes and changes in cerebral blood flow [61]; secondly, a ‘forced normalisation’ process whereby an inverse relationship exists between seizure control and psychotic symptoms [57,62] and thirdly, on-going subictal activity in the limbic system that is undetectable on EEG but which leads to brain changes that result in psychosis [55,63].

Antiepileptic medication may also play a role in the development of psychosis especially among patients with other risk factors such as family history or past psychiatric history. Psychoses have been noted as a potential adverse effect in many different antiepileptic medications suggesting that the phenomenon is not medication specific. Antiepileptic medications implicated include ethosuxamide, topiramate, vigabatrin, zonisamide and leviteracetam [64]. One study found a prevalence of psychosis of 3.7% in patients after they were commenced on topiramate [65]. High starting doses of medication and a rapid titration schedule in more vulnerable patients with past psychiatric history and with more severe epilepsy with high seizure frequency were associated with greater risk.

However, it is also possible that epilepsy and psychotic illness may represent different outcomes of a common aetiological process. Neuropathological, neuroimaging and genetics findings show that similar structural brain abnormalities and genetic abnormalities are present in patients with schizophrenia and patients with epilepsy [55,66-71]. For instance, enlarged ventricles have been found to be common to first episode psychosis and temporal lobe epilepsy patients without psychosis [67]. Neuronal migration defects have been proposed as a mechanism related to enlarged ventricles and this defect could be common both to schizophrenia and epilepsy. From a neurobiological perspective, significant grey and white matter deficits occur in temporal lobe epilepsy with psychosis. Some of these deficits overlap with those found in schizophrenia. These include the medial temporal structures but also extend to lateral temporal and extratemporal regions [70].

Recent genetic work shows that a rare genetic mutation can lead to either epilepsy or schizophrenia. A micro-deletion in the genomic area 15q 13–14 containing the nicotine receptor was linked to development of either schizophrenia or juvenile epilepsy [68,69,71]. The gene leucine-rich glioma-inactivated 1 gene (LGI1) in autosomal dominant partial epilepsy with auditory features, [72,73] may also play a role in regulating glutaminergic synaptic transmission, a process that is involved in the pathophysiology of schizophrenia [74]. Genes encoding ion channels may also be a source of interest. Ion channelopathies are known to underlie some epilepsies and it has been shown that variation within the gene CACNA1C (encoding a subunit of the L-type voltage dependent calcium channel) is associated with schizophrenia as well as depression and bipolar affective disorder [75]. This study provides support for the possibility that some people might experience both psychosis and epilepsy at least in part because of an underlying vulnerability to both.

## Conclusions

In summary, adequate recognition and treatment of psychosis in epilepsy is essential for patient management because of their considerable burden in morbidity and quality of life [12,76]. We would recommend that future studies in this area should be used for defining psychotic presentations. Improved diagnostic classification would allow better characterisation of prevalence rates. Unfortunately standardized criteria for psychiatric disorders such as ICD 10 or DSM V do not allow this presently. Further investigations in population-based studies are warranted for a more accurate prevalence rate at a population level and family-based studies are needed to investigate the possible clustering of psychosis and epilepsy within families. In conclusion, this study is the first meta-analysis and systematic review on the prevalence of psychosis in epilepsy. The methodological rigour of a systematic review adds clarity to the previous findings on this topic and confirms the results. We report an almost 8-fold increased risk of psychosis in epilepsy. An improved understanding of the mechanisms underlying this association would be a fruitful line of enquiry and may yield useful information on the aetiopathogenesis of both psychosis and epilepsy.

## Competing interests

The authors declare that they have no competing interests.

## Authors’ contributions

MJC and DRC conceived the idea for the study. MJC and DJC were responsible for the data collection. MCC was responsible for all the statistical analysis. MJC wrote the first draft of the paper. MJC, MCC, MC and DRC all reviewed and edited the manuscript. MJC, MCC, MC and DRC revised it critically. All authors approved the final version of the manuscript.

## Authors´ information

Mary Cannon and David Cotter are joint senior author contributors.

## Pre-publication history

The pre-publication history for this paper can be accessed here:

http://www.biomedcentral.com/1471-244X/14/75/prepub
